# Anti-apoptotic ARC protein confers chemoresistance by controlling leukemia-microenvironment interactions through a NFκB/IL1β signaling network

**DOI:** 10.18632/oncotarget.7911

**Published:** 2016-03-04

**Authors:** Bing Z. Carter, Po Yee Mak, Ye Chen, Duncan H. Mak, Hong Mu, Rodrigo Jacamo, Vivian Ruvolo, Stefan T. Arold, John E. Ladbury, Jared K. Burks, Steven Kornblau, Michael Andreeff

**Affiliations:** ^1^ Section of Molecular Hematology and Therapy, Department of Leukemia, The University of Texas MD Anderson Cancer Center, Houston, TX, USA; ^2^ King Abdullah University of Science and Technology (KAUST), Computational Bioscience Research Center, Division of Biological and Environmental Sciences and Engineering, Thuwal, Saudi Arabia; ^3^ Department of Biochemistry and Molecular Biology and Center for Biomolecular Structure and Function, The University of Texas MD Anderson Cancer Center, Houston, TX, USA

**Keywords:** AML, ARC, NFκB, chemoresistance

## Abstract

To better understand how the apoptosis repressor with caspase recruitment domain (ARC) protein confers drug resistance in acute myeloid leukemia (AML), we investigated the role of ARC in regulating leukemia-mesenchymal stromal cell (MSC) interactions. In addition to the previously reported effect on AML apoptosis, we have demonstrated that ARC enhances migration and adhesion of leukemia cells to MSCs both *in vitro* and in a novel human extramedullary bone/bone marrow mouse model. Mechanistic studies revealed that ARC induces IL1β expression in AML cells and increases CCL2, CCL4, and CXCL12 expression in MSCs, both through ARC-mediated activation of NFκB. Expression of these chemokines in MSCs increased by AML cells in an ARC/IL1β-dependent manner; likewise, IL1β expression was elevated when leukemia cells were co-cultured with MSCs. Further, cells from AML patients expressed the receptors for and migrated toward CCL2, CCL4, and CXCL12. Inhibition of IL1β suppressed AML cell migration and sensitized the cells co-cultured with MSCs to chemotherapy. Our results suggest the existence of a complex ARC-regulated circuit that maintains intimate connection of AML with the tumor microenvironment through NFκB/IL1β-regulated chemokine receptor/ligand axes and reciprocal crosstalk resulting in cytoprotection. The data implicate ARC as a promising drug target to potentially sensitize AML cells to chemotherapy.

## INTRODUCTION

Acute myeloid leukemia (AML) is a hematological malignancy with poor long-term survival primarily due to the development of chemoresistance. Multiple leukemia-intrinsic mechanisms of chemoresistance have been established, but the clinical experience of AML treatment also suggests that circulating leukemic cells are strikingly more sensitive to chemotherapy than their bone marrow (BM) counterparts. This finding has motivated the field to explore mechanisms that keep AML cells tethered to the protective BM niche and develop therapies to disrupt leukemia-niche interactions.

BM-derived mesenchymal stromal cells (MSCs) are an essential structural and functional component of the BM microenvironment, and they are critical for hematopoiesis [1]. Within the context of leukemia, MSCs also play an essential role in protecting leukemia cells from chemotherapeutic agents [2]. Much of the interaction occurs through a wide range of adhesion molecules on hematopoietic cells and their corresponding ligands secreted by BM stromal cells. While this system of communication is typically used between normal hematopoietic cells and BM stromal cells, it is hijacked by leukemic cells to enhance interactions that protect these cells from chemotherapy [3–7].

CXCR4/CXCL12 is perhaps the best understood receptor/ligand axis involved in AML. Elevation of CXCR4 in AML cells and CXCL12 (also known as SDF1α) in MSCs have both been detected in AML patient samples, with the former associating with poor disease prognosis [8–10]. Increased expression and activity of CXCR4 and CXCL12 have also been shown to enhance tumor invasiveness, growth, and metastasis in several other types of cancer [11, 12]. Further, pharmacological blockade of the CXCR4/CXCL12 axis results in chemosensitization of AML cells *in vitro* and *in vivo*, therefore serving as a biological basis for development of various CXCR4 and CXCL12 inhibitors [13–16]. However, this strategy has only shown limited success in mobilizing AML cells from the BM microenvironment clinically, possibly in part due to parallel activity of other chemokine/receptor axes that also contribute to leukemia-stromal interactions.

In addition to the protection by BM MSCs, genes that regulate apoptosis are frequently deregulated in leukemic cells, which further supports their survival during chemotherapy. Hence, simultaneous targeting of apoptosis regulators and leukemia-stromal interactions could be a novel strategy to overcome the development of chemoresistance and disease relapse in AML [2, 17]. One such regulator, the apoptosis repressor with caspase recruitment domain (ARC) protein, is capable of suppressing both intrinsic and extrinsic apoptosis via inhibiting Fas/FADD interaction, blocking caspase-2/-8 activity, and/or interfering p53 and Bax function [18–22]. Increased ARC expression was recently shown to occur in various cancer cell types, correlating with poorer disease prognosis, and conferring chemo- and radio-resistance in transformed cells [18, 23–26]. Our previous research, using reverse-phase protein array (RPPA) on 511 newly diagnosed AML samples, implicated that high ARC expression correlated strongly with a poor survival in AML patients [27]. Additionally, we elucidated that ARC expression is upregulated via MAPK/PI3K signaling activated by MSCs, an interaction that confers drug resistance and a survival advantage to these AML cells [28]. We also identified that ARC suppresses AML cell death by antagonizing p53 and suppressing TRAIL [29].

While it is clear that ARC contributes to AML chemoresistance and is associated with poor prognosis, the precise molecular mechanisms that link this anti-apoptotic protein to such a phenotype are still to be fully elucidated. In the aforementioned RPPA analysis, we also assessed the expression of over 200 proteins likely associated with AML pathobiology. We found that enhanced ARC expression correlated with elevated levels of numerous proteins that are involved in cell signaling, adhesion, and migration. This prompted us to investigate the role of ARC in leukemia-stromal interactions. Here we reveal that ARC mediates a complex regulatory circuit likely via NFκB/IL1β signaling in both AML cells and MSCs, leading to activation of numerous chemokine ligand/receptor axes that foster their close association and leukemic cell chemoresistance, therefore a novel target for AML.

## RESULTS

### ARC both in AML cells and MSCs regulates leukemia-stromal interactions

**Figure 1:**
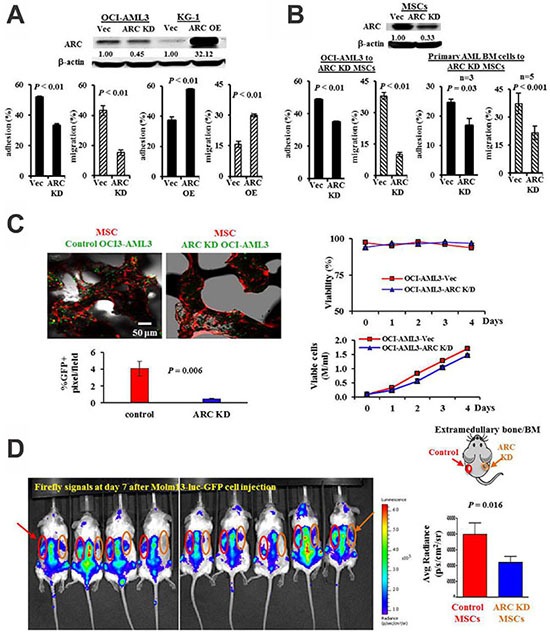
ARC regulates leukemia-stromal interactions (**A**) Adhesion and migration of ARC KD OCI-AML3 and ARC OE KG-1 or their control cells to MSCs. (**B**) Adhesion and migration of OCI-AML3 and BM cells from AML patients to ARC KD or control MSCs. Adhesion was determined at 24 h and migration at 6 h for OCI-AML3 and 24 h for patient samples. (**C**) ARC KD or control OCI-AML3 cells (expressing GFP) were co-cultured with MSCs (expressing RFP) growing on the surface of bone chips. Attachment of leukemia cells to MSCs was measured at 24 h by confocal microscopy. A representative image and the quantifications from four images are shown on the left and viability and growth curves of ARC KD and control OCI-AML3 cells are shown on the right. (**D**) Molm13 cells (1 × 10^6^) stably expressing a dual *firefly* luciferase–GFP reporter were injected into the tail vein of NSG mice harboring extramedullary bone/BM developed from either ARC KD or control human MSCs on each flank. Leukemia burden was monitored by IVIS imaging. Vec, vector control.

Cell adhesion and migration assays were performed first using stable ARC knockdown (KD) OCI-AML3 (high endogenous ARC expression) or overexpression (OE) KG-1 (low endogenous ARC expression) AML cells. The ARC KD OCI-AML3 cells exhibited less adhesion to and migration toward MSCs than their vector controls. Conversely, the ARC OE KG-1 cells exhibited more adhesion to and migration toward MSCs than the vector controls (Figure 1A). Next, we knocked down ARC in MSCs and queried whether this would affect adhesion and migration of AML cells. Indeed, fewer cells from both an AML cell line and primary patient BM samples adhered (*n* = 3) and migrated (*n* = 5) to the ARC KD MSCs compared to the control MSCs (Figure 1B).

We further assessed AML cell adhesion using a bone chip model, which provides a three-dimensional scaffold for MSC growth, mimicking *in vivo* structural dynamics. MSCs expressing RFP were grown on the bone chip surface and co-cultured with ARC KD or control OCI-AML3 cells expressing GFP. We found significantly fewer GFP positive pixels (an 88% decrease, *P* = 0.006) on bone chip associated MSCs cultured with ARC KD OCI-AML3 cells relative to those cultured with control OCI-AML3 cells (Figure 1C left panel, representative image and quantification of 4 images). Although ARC knockdown sensitizes AML cells to chemotherapeutic agents [28], it neither altered AML cell viability nor markedly decreased cell growth (Figure 1C right panel), suggesting that decreased association of ARC KD OCI-AML3 cells to MSCs resulted from a decreased adhesion property in these cells. Finally, we investigated the role of ARC in MSCs using the *in vivo* human extramedullary bone/BM model [30]. ARC KD or control human MSCs and human endothelial colony-forming cells (ECFC) (1:1) were mixed with matrigel and injected into the right or left flank of NOD/SCID IL2Rg null (NSG) mice, respectively (Figure 1D). Once the bone was established, GFP/luciferase-labeled Molm13 cells were administered by tail vein injection. Significantly fewer (48.3% decrease, *P* = 0.016 at 7 days) leukemia cells engrafted per cm^2^ in the human extramedullary bone/BM constituted with ARC KD MSCs versus with the control MSCs (Figure 1D). Collectively, these results indicate that ARC expression in both AML cells and MSCs mediates interactions between these cells.

### ARC regulates CXCL12, CCL2, and CCL4 expression in MSCs, supporting AML cell chemotaxis

**Figure 2:**
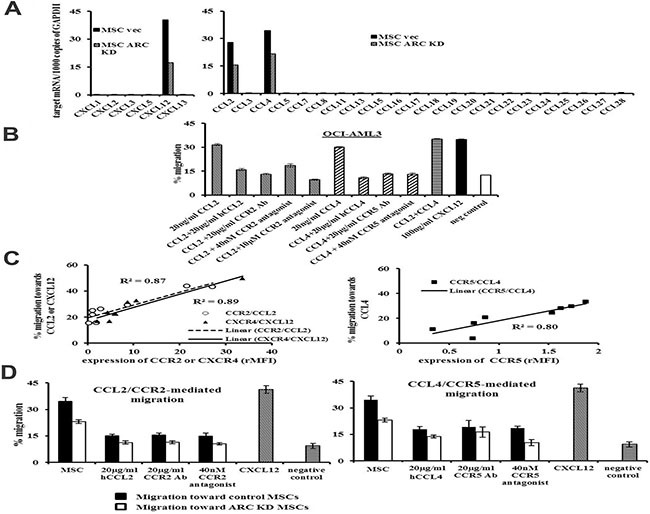
ARC regulates CXCL12, CCL2, and CCL4 production in MSCs and promotes chemokine-mediated leukemia-stromal interactions (**A**) Expression of various C-X-C and C-C motif chemokines in ARC KD and control MSCs determined by quantitative PCR array. (**B**) Chemotaxis of OCI-AML3 cells to CCL2 and CCL4 in the presence or absence of antibodies or inhibitors against CCL2, CCR2, CCL4, and CCR5 for 4 h. (**C**) Correlation of CCR2, CCR5, and CXCR4 expression in BM cells from patients with AML (*n* = 8) and migrations to CCL2, CCL4, and CXCL12. (**D**) Migration of OCI-AML3 to ARC KD or control MSCs in the presence or absence of antibodies or inhibitors against CCL2, CCR2, CCL4, and CCR5 for 6 h. Chemotaxis to CXCL12 (100 ng/ml) was used as positive and random migration as negative controls. hCCL2 and hCCL4, neutralizing antibodies for CCL2 and CCL4, respectively.

To better understand the mechanism(s) of the ARC-regulated leukemia-stromal interactions, we determined the expression of several chemokines in ARC KD, ARC OE, and their respective control cells by PCR array. Among the various C-X-C and C-C motif chemokines tested, CXCL12, CCL2, and CCL4 were expressed at high levels in MSCs, and their expression was greatly reduced when ARC was knocked down in MSCs (Figure 2A). Minimal levels of these chemokines were detected in AML cells. While it was known that OCI-AML3 cells migrate toward CXCL12, a migration assay showed that these cells also migrated toward CCL2 and CCL4. This migratory activity was inhibited by anti-CCR2 and CCR5 antibodies and small molecule inhibitors that antagonize or compete with CCL2 and CCL4 (Figure 2B). Further, CCL2, CCL4, or CXCL12 induced the migration of cells from eight AML patient BM samples, and this chemotaxis positively correlated with expression of the respective receptors for these cytokines on leukemic cells from these samples (Figure 2C). ARC KD in the MSCs partially suppressed the migration of OCI-AML3 cells, and migration was further suppressed by antibodies and small-molecule antagonists against CCL2/CCR2 or CCL4/CCR5 (Figure 2D).

**Figure 3:**
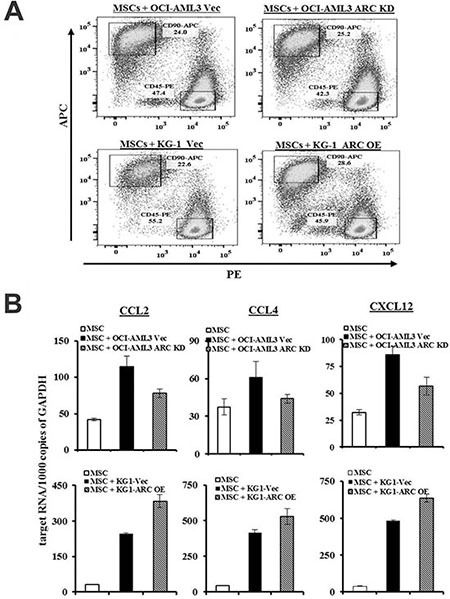
ARC in AML modulates CCL2, CCL4, and CXCL12 expression in MSCs (**A**) MSCs were cultured alone or with ARC KD OCI-AML3, ARC OE KG-1, or the respective control cells for 48 h and MSCs were FACS sorted conservatively as marked in the boxes for CD45^−^CD90^+^ cells. (**B**) CCL2, CCL4, and CXCL12 levels in MSCs were determined by quantitative RT-PCR.

Next, we sought to determine if MSC chemokine expression was affected by exposure to leukemic cells. We co-cultured MSCs and OCI-AML3 cells for 48 h and then FACS-sorted the MSCs (CD45^−^90^+^) from the AML cells (CD45^+^90^−^) with conservative gating (Figure 3A). Interestingly, co-culture induced CCL2, CCL4, and CXCL12 expression in the MSCs. This induction was diminished when MSCs were co-cultured with ARC KD AML cells, and increased in the presence of ARC OE AML cells (Figure 3B). Collectively, these results suggest that like CXCR4/CXCL12, the CCR2/CCL2 and CCR5/CCL4 receptor/chemokine axes contribute to leukemia-MSC interactions, and that the chemokines expressed by MSCs are regulated, at least in part, by ARC in AML cells.

### ARC-mediated IL1β activation in AML cells induces chemokine production in MSCs that confer leukemia cell chemoresistance

**Figure 4:**
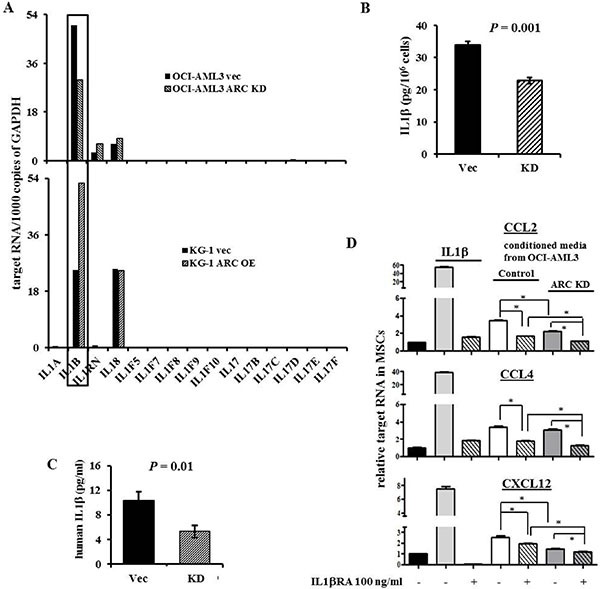
ARC regulates IL1β in AML cells and triggers chemokine productions by MSCs (**A**) Cytokine expressions in ARC KD, ARC OE, and the control AML cells determined by real time RT-PCR. (**B**) The expression of IL1β in the supernatant of ARC KD and control OCI-AML3 cells determined by ELISA. (**C**) The expression of human IL1β in the serum of NSG mice three weeks after the mice were injected with ARC KD or control Molm13 cells determined by ELISA. (**D**) The expression of CCL2, CCL4, and CXCL12 in MSCs treated with IL1β (100 ng/ml) or conditioned medium from control or ARC KD OCI-AML3 cells with or without IL1βRA for 24 h. *P < 0.05.

We next examined cytokine expression by PCR array in ARC KD and ARC OE cells to possibly gauge ARC's role in the regulation of these cytokines. Interestingly, IL1β was overexpressed in the ARC OE AML cells and reduced in ARC KD AML cells compared to respective vector control cells (Figure 4A). The same assay found minimal levels of IL1β in MSCs. Conversely, the IL1 receptor antagonist protein, IL1RN, was higher in ARC KD, and lower in ARC OE AML cells, than observed in control cells (Figure 4A). As IL1RN levels were very low in these cells, we focused on the IL1β. Protein analysis by ELISA showed that ARC KD OCI-AML3 cells secreted less IL1β than their respective controls (Figure 4B). Furthermore, NSG mice injected with ARC KD Molm13 cells had approximately half of human IL1β concentration in their serum (Figure 4C), while the leukemia burden was about 30% less in these mice compared to the mice injected with control Molm13 cells after three weeks [28], suggesting that ARC may exert some of its function through IL1β signaling.

To test this hypothesis, we treated MSCs with IL1β or conditioned medium from AML cells with or without an IL1β receptor antagonist (IL1βRA). Indeed, IL1β alone or the conditioned medium from AML cells increased the level of CCL2, CCL4, and CXCL12 expression in MSCs. This effect was diminished when the conditioned medium from ARC KD AML cells was used, and was further suppressed by co-treating the MSCs with IL1βRA (Figure 4D).

**Figure 5:**
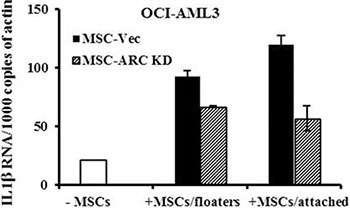
MSCs induce the expression of IL1β in AML cells OCI-AML3 cells were cultured alone or co-cultured with ARC KD or control MSCs for 48 h. CD45^+^CD90^−^ AML cells were FACS-sorted from the floaters (collected from cells in the suspension and after PBS wash) and attached (collected by trypsinization) cells. IL1β RNA levels were determined in sorted OCI-AML3 cells and the cells cultured alone by real-time RT-PCR.

To assess if cytokine expression in leukemia cells was also regulated by MSCs, OCI-AML3 cells were co-cultured for 48 h with MSCs, sorted as shown in Figure 3A, and their IL1β expression was determined by real-time RT-PCR. We found that co-culture increased the expression of IL1β in AML cells. This effect was reduced, compared to the control MSCs, when the leukemic cells were co-cultured with ARC KD MSCs (Figure 5).

**Figure 6:**
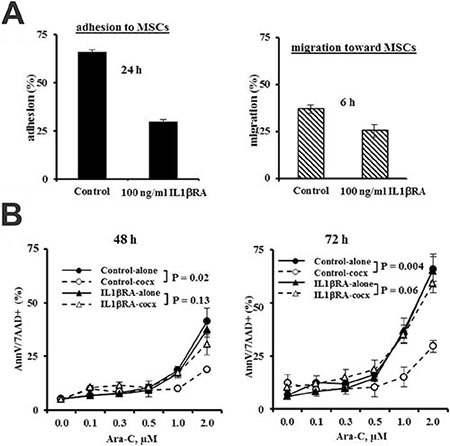
Inhibition of IL1β suppresses adhesion and chemotaxis of leukemia cells to MSCs and abolishes chemoprotection of MSCs to leukemia cells (**A**) OCI-AML3 cells were added to MSCs which were set the night before and treated with ILβRA (100 ng/ml). Cell adhesion was determined after 24 h. OCI-AML3 cells were pre-treated with ILβRA (100 ng/ml) for 1 h and their migration towards MSCs was determined at 6 h. (**B**) OCI-AML3 cells, cultured alone or with MSCs were treated with Ara-C in the presence or absence of ILβRA (100 ng/ml). Apoptosis was assessed at 48 and 72 h by flow cytometry after staining cells with annexin V (AnnV) and 7-aminoactinomycin D (7AAD).

To determine the biological consequence of IL1β blockade in leukemia/stromal interactions, we next performed an adhesion/migration assay and found that inhibition of IL1β by IL1βRA suppressed the adhesion/migration of leukemia cells to MSCs (Figure 6A). We previously demonstrated that ARC KD AML cells are more sensitive and ARC OE AML cells more resistant to various therapeutic agent-induced apoptosis including Ara-C [28]. To determine whether ARC-regulated IL1β, which has a role in leukemia-stromal interactions, also impacts chemosensitivity, we next treated OCI-AML3 cells with Ara-C in the absence or presence of MSCs and/or IL1βRA. As expected, the presence of MSCs significantly (*P* = 0.02 and 0.004, respectively) protected the leukemia cells from Ara-C-induced cell death at 48 and 72 h (Figure 6B). Inhibition of IL1β had little effect on Ara-C-induced apoptosis when OCI-AML3 cells were cultured alone. However, blocking IL1β by IL1βRA markedly abolished the protection of OCI-AML3 by MSC co-culture following a similar exposure to Ara-C (Figure 6B, *P* = 0.13 and 0.06, respectively).

### ARC regulates NFκB signaling in leukemia cells and MSCs

**Figure 7:**
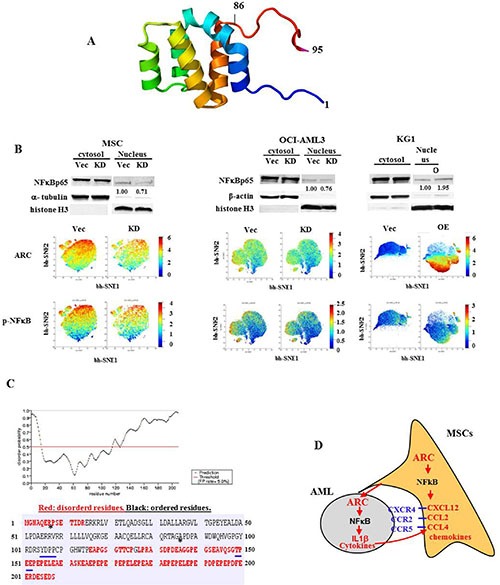
ARC regulates NFκBp65 signaling (**A**) N-terminal region of ARC adopts an atypical CARD domain fold where the sixth helix is unfolded. The model shown is color-ramped from the N-terminus (blue, residue 1) to the C-terminus (red, residue 95). The program SwissModel was used to model residues 87-90 which are not included in the PDB model (entry 4UZ0). (**B**) The expression levels of NFκBp65 protein in cytoplasmic/nuclear fractions determined by western blot and the expression levels of p-NFκBp65 and ARC proteins by CyTOF in ARC KD and OE cells. (**C**) The C-terminal of ARC is most likely disordered. YDPP (positions 105–108) is a SH2-domains binding motif and TPEE (positions 149–152) is a TRAF2-binding consensus motif (both underlined with blue). Asterisks (*) identify the start and end of the stably folded CARD domain of ARC in the crystallographic structure 4UZ0. (**D**) A putative mechanism of ARC-regulated leukemia-stromal interactions through NFκB/IL1β signaling.

Aberrant expression of caspase activation and recruitment domain (CARD) proteins has been implicated in the etiology of various cancers [31]. These proteins have diverse functions, such as inducing IL1β by caspase-1-mediated cleavage and activating NFκB [31, 32]. Our results have shown that ARC transcriptionally induces the expression of IL1β. Given that ARC regulates the expression of multiple NFκB-targeted cytokines/chemokines including IL1β and CCL2 (Figures 2 and 4), this suggests that ARC might exert this activity via activation of NFκB. Our computational three-dimensional structure analysis (Figure 7A) and the recently published experimental structure of ARC residues 1–95 [33] corroborated that the N-terminal domain of ARC adopted a CARD domain fold.

We first compared the expression of ARC and NFκB in our RPPA data set and did not find a correlation between ARC and NFκB protein expression. This was not surprising since the NFκB determined in AML samples by RPPA was the total, not the active form (nuclear localization/phosphorylated) of the protein. We next determined the cytosolic and nuclear protein levels of NFκBp65 and found that nuclear NFκBp65 levels are lower in ARC KD MSCs and OCI-AML3 cells and higher in ARC OE KG-1 cells than controls (Figure 7B, upper panel). We then examined ARC and p-NFκBp65 protein levels by CyTOF mass cytometry and found that p-NFκBp65 levels are lower in ARC KD MSCs and OCI-AML3 cells and higher in ARC OE KG-1 cells compared to controls. In the cells that had high levels of ARC protein there was also a high expression of the p-NFκBp65 protein (Figure 7B, lower panel). Although nuclear NFκBp65 and p-NFκBp65 expression increased in ARC OE KG-1 cells, the increase was much less in magnitude compared with the increase in ARC levels, suggesting that ARC was possibly interacting with other co-factor(s) to activate NFκB. This is supported by the structural analysis showing that the disordered C-terminal of ARC (Figure 7C) contains several-binding consensus motifs such as a TRAF2-binding motif (TPEE, positions 149–152) and a SH2-domain binding motif (YDPP, positions 105–108) (Figure 7C, underlined in blue). It may also reflect that NFκB is regulated at multiple levels.

## DISCUSSION

The anti-apoptotic protein ARC may have cellular roles beyond apoptosis regulation. For example, it has been reported to promote breast tumorigenesis, metastasis, and chemoresistance [34], and was identified in a microarray analysis as a predictor of invasion and metastasis in human cancers [35]. However, the mechanism of action has not been elucidated. The results presented in this report support these findings, and importantly, demonstrate that ARC regulates leukemia/stromal interactions likely via several NFκB/IL1β-mediated receptor/chemokine axes that seem to further support AML cell chemoresistance.

We first demonstrated that AML cells migrate toward and associate with MSCs in an ARC-dependent manner *in vitro*, then *in vivo* using a human extramedullary BM mouse model [30, 36]. This model allowed us to genetically modulate genes of interest in the human extramedullary microenvironment. Using this model, we showed that ARC in MSCs is required for full leukemia engraftment. The results strongly support the role of ARC in MSCs as a major regulator of microenvironmental interactions.

Having established the importance of ARC in AML cell-MSC interactions, we queried chemokine/cytokine expression in both MSCs and leukemic cells to determine if these cells exchange chemical signals to mediate such interactions. We discovered that chemokines CCL2, CCL4, and CXCL12 are regulated in MSCs in an ARC-dependent manner, their cognate receptors CCR2, CCR5, and CXCR4 were expressed in AML cells, and that inhibition of receptor/ligand interactions blocked AML cell migration. Next, we observed that the expression of chemokine in MSCs was also affected by ARC expression in the leukemic cells. Further investigation led us to IL1β, a cytokine that is upregulated in AML cells in an ARC-dependent manner and also appears to stimulate production of the same three cytokines from MSCs. Additionally, IL1β expression in leukemic cells was increased by co-culture with MSCs, and neutralization of IL1β signaling by antagonizing IL1R reduced migration and adhesion of AML cells to MSCs. Our observation that MSCs protected AML cells from killing by chemotherapeutic compound Ara-C, but diminished when IL1β was blocked, allowed us to conclude that this circuitry is crucial for chemoprotection of AML cells by MSCs. Collectively, these results strongly suggest a model of reciprocal interaction by which ARC in both MSCs and AML cells promotes expression of chemokines via IL1β that foster a tight association between these two cell types and confer AML cell chemoprotection (Figure 7D).

We also implicate NFκB as an intermediate player in this network, showing that levels of nuclear/activated NFκB decrease in both ARC knockdown MSCs and AML cells. Our results suggest that ARC may activate NFκB in both cell types, leading to upregulation of IL1β in AML cells and CCL2, CCL4, and CXCL12 in MSCs. It is already established that IL1β is a transcriptional target of NFκB [37], and that ARC contains a CARD domain typical of NFκB-activating proteins [38]. Interestingly, when ARC was overexpressed in KG-1 cells, we did not see a proportional induction of NFκB activation and IL1β expression or increase in cell migration/adhesion. This could be because ARC may have other interaction partners required for full NFκB activation due to the presence of other regulatory binding domains, such as TRAF2, within the ARC protein sequence.

We identify that, in addition to the well-known CXCR4/CXCL12 axis, CCR2/CCL2 and CCR5/CCL4 also drive leukemia/stromal interactions. Specifically, CCL2 is one of the most highly expressed chemokines in BM-derived MSCs, and its expression is regulated through ARC in MSCs and by ARC-induced IL1β from leukemia cells. Further, the CCL2 receptor, CCR2 was found to be highly expressed on BM cells from AML patients, and migration of AML blasts towards CCL2 strongly correlated with their CCR2 expression. Similar results were observed for CCL4 and its receptor CCR5, albeit the CCR5 expression was lower than CCR2 in the AML cells. This is the first report on the important role of ARC in the regulation of the CCR2/CCL2 and CCR5/CCL4 axes in leukemia and stromal cells. These data may explain, at least in part, why the disruption of CXCR4/CXCL12 axis alone has limited effect in the mobilization of leukemia cells, including leukemic stem cells, from their BM microenvironment.

IL1β has previously been linked to AML, promoting apoptosis-resistance in AML blasts [39], and the IL-1 receptor accessory protein (IL1RAP) is reportedly overexpressed in AML stem/progenitor cells [40]. Furthermore, targeting of IL1RAP with a neutralizing antibody selectively killed AML stem cells [41]. These data support the critical role of IL1β signaling in AML cell survival in the BM microenvironment. Likewise, NFκB is constitutively activated in primitive AML cells [42], and it reportedly induces VCAM-1 expression to regulate MSC accumulation at tumor sites [43]. Furthermore, VCAM-1/VLA-4 mediated leukemia-stromal interactions activate NFκB, which confers chemoresistance [36]. It is possible that additional chemokines, cytokines, and microRNAs are also important for ARC-mediated leukemia/stromal interactions and chemoresistance in AML cells, which warrants further investigation. The results reported here can explain in part the diverse biological functions of ARC, and suggest that therapeutic inhibition of this protein potentially represents a novel approach to the treatment of AML as an adjuvant to standard chemotherapy.

## MATERIALS AND METHODS

### Cell culture

OCI-AML3 cells were provided by Dr. M. Minden (Ontario Cancer Institute, Toronto, Ontario, Canada). KG-1 cells were purchased from the American Type Culture Collection (ATCC, Manassas, VA), and Molm13 cells were purchased from the German Collection of Microorganisms and Cell Cultures (Braunschweig, Germany). Stable ARC KD OCI-AML3 cells and ARC OE KG-1 cells were generated as described [28]. Early-passage MSCs were isolated from the BM of healthy subjects [44], and stable ARC KD MSCs were generated as described previously [45]. Briefly, ARC was knocked down in OCI-AML3 and MSCs by lentiviral transduction using gene-specific shRNAmir-green fluorescent protein (GFP)-expressing transfer vectors: clone V3LHS_337663, targeting residues 732-750 on RefSeq NM_003946.4 (Open Biosystems, Huntsville, AL). Lentivirus was prepared by co-transfection of HEK293T cells (ATCC) with an equal molar mix of transfer vector and packaging plasmids (psPAX2 and pMD2.G; Addgene, Cambridge, MA) using JetPrime transfection reagent as directed by the manufacturer (Polyplus, Illkirch, France). Fresh lentiviral supernatants were passed through 0.45-micron-pore surfactant-free cellulose acetate membranes and then used immediately to infect OCI-AML3 cells or MSCs. Infected cells were subjected to selection with puromycin (Invivogen, San Diego, CA) starting at 1 μg/ml. As controls, OCI-AML3 cells and MSCs were transduced with lentivirus delivering a non-specific control vector (Open Biosystems). To generate ARC OE KG-1 cells, the ARC coding sequence was excised from EGFP-Myp (kindly provided by Dr. S. Stamm, University of Kentucky, Lexington, KY) with *Mlu*I-*Bgl*II and its ends filled in with Klenow before it was cloned into pCDH-CMV-MCS-EF1-copGFP (SystemBio, Mountain View, CA) between the blunted *Nhe*I-*Not*I sites. The resulting lentiviral vector was designated pCDH-CMV-ARC-EF1-copGFP. KG-1 cells were infected with concentrated lentivirus transduced with either pCDH-CMV-ARC-EF1-copGFP or pCDH empty vector generated by a process similar to that just described; 8 μg/ml Polybrene (Sigma Chemical Co., St. Louis, MO) was included to enhance lentiviral infections. Stably transduced KG-1 cells were sorted to obtain a homogeneous population of ARC OE CopGFP-positive cells. KD and OE were verified by western blot analysis and by real-time RT-PCR.

Fresh BM samples from high blast AML patients were acquired after written, informed consent according to the Declaration of Helsinki. The study protocol was approved by The University of Texas MD Anderson Cancer Center Institutional Review Board. Mononuclear cells were purified from the patient samples using a Ficoll-Hypaque (Sigma-Aldrich, St. Louis, MO) density-gradient centrifugation. All cells were cultured in RPMI 1640 medium supplemented with 10% heat-inactivated fetal calf serum, 2 mM L-glutamine, 100 U/ml penicillin, and 100 μg/ml streptomycin.

OCI-AML3 cells were treated with Ara-C, with or without MSC co-culture and with or without IL1βRA for 72 h. MSCs were treated with IL1β or a conditioned medium for 24 h. Recombinant IL1βRA and ILIβ were purchased from PeproTech (Rocky Hill, NJ).

### Adhesion assay

For MSC adhesion, leukemia cells were added to MSCs (AML cells: MSCs = 4:1) that were plated the night before and cultured for 24 h in 5% CO_2_ at 37°C. Floating leukemia cells (in suspension plus collected after a wash with phosphate-buffered saline) and attached leukemia cells (collected by trypsinization) were counted by flow cytometry after staining with human CD45 antibody in the presence of counting beads (purchased from Life Technologies, Grand Island, NY). Adhesion was defined as viable CD45^+^ attached cells/total viable leukemia cells (floating + attached CD45^+^ cells).

For the three-dimensional MSC-bone chip adhesion assay, cancellous bone chips (two to four chips, 2 mm^3^, purchased from Medtronic, Minneapolis, MN) were incubated with RFP-expressing MSCs (2 × 10^6^ in 1 ml) overnight in 5% CO_2_ at 37°C. Bone chips/MSCs were washed with medium to remove the unattached cells and then placed in tissue culture plates. GFP-expressing OCI-AML3 cells were added. The mixtures were incubated overnight in 5% CO_2_ at 37°C. The chips were gently washed with medium to remove unattached leukemia cells. Images were captured using an Olympus confocal microscope FV1000 at 100× magnification and analyzed using InForm 2.0 (Perkin Elmer, Waltham, MA) software. A threshold for positivity was set and the pixels above threshold were counted. The positive pixels were then summed and compared with four images from each group to determine expression differences.

### Migration assays

MSCs (5000 cells/ml) were plated in the lower chamber of trans-well plates (5 μm) and cultured for 24 h. Leukemia cells, 0.4 × 10^6^ (2 × 10^6^/ml), were added in the insert and cultured for 6 to 24 h. The percentage of migration was defined as viable CD45^+^ cells in the lower chamber/total viable leukemia cells in insert and lower chamber. For migration toward ligands, chemokines were added to the lower chamber and the migration of leukemia cells was measured at 4 to 6 h. CCL2, CCL4, and CXCL12 ligands, neutralizing antibodies for CCL2 (hCCL2) and CCL4 (hCCL4) and antagonist for CCR5 (maraviroc) were purchased from R & D Systems Inc. (Minneapolis, MN). Neutralizing antibodies for CCR2 and CCR5 were purchased from Abcam (Cambridge, MA), and antagonist for CCR2 was purchased from Santa Cruz Biotechnology, Inc. (Dallas, TX).

### Cell viability assay

Apoptosis was assessed by phosphatidylserine externalization after annexin-V-Cy5 (BD Biosciences) staining using a FACSArray Bioanalyzer (BD Biosciences) flow cytometer. Cell membrane integrity was simultaneously assessed by 7-aminoactinomycin D exclusion in the annexin V-stained cells. For AML cells co-cultured with MSCs, cells were trypsinized and stained with CD45-APC-H7, CD90-PE, and annexin-V-Cy5. Apoptosis in AML cells was determined by flow cytometric analysis of annexin-V-Cy5 positivity in CD45^+^CD90^−^ cells.

### *In vivo* study

The *in vivo* human leukemia-stromal interaction study was carried out using a human extramedullary bone/BM model in NSG mice developed and validated by our group [30, 36]. Briefly, ARC KD or control human MSCs and human ECFC (1:1) were mixed with matrigel (Millipore, Billerica, MA) then injected subcutaneously into flanks of NSG mice. 10-week later, 1 × 10^6^ Molm13 cells expressing a dual firefly luciferase–GFP reporter were transplanted into mice by tail vein injection. Tumor burden was monitored using the IVIS-200 noninvasive bioluminescence *in vivo* imaging system (Xenogen, Hopkinton, MA) after injecting the mice with luciferin.

### Fluorescence-activated cell sorting (FACS)

AML cells (4 × 10^6^ at 0.2 × 10^6^/ml) were added to MSCs (1 × 10^6^ at 5000 cells/cm^2^) plated the night before and co-cultured for 48 h. CD45^+^/CD90^−^ leukemia cells and CD45^−^CD90^+^ MSCs were FACS-sorted (FACS Aria II, BD Biosciences, San Jose, CA) after trypsinization and stained with CD45 and CD90 antibodies and subjected to RNA isolation and real time RT-PCR.

### RT-PCR

RNA isolation and RT-PCR were performed as previously described [46] with minor modifications. The PCR amplification mixture (20 μl) contained cDNA, a primer pair of human cytokine primer library II primers for pathway PCR array (Real Time Primers, LLC, Elkins Park, PA), and SYBR Green PCR master mix (Applied Biosystems, Foster City, CA). The abundance of each transcript relative to that of GAPDH or actin was calculated using the 2^−ΔCt^ method, where ΔCt is the mean Ct of the transcript of interest minus the mean Ct of the transcript for GAPDH or actin.

### Protein expression and localization

Western blot analysis was performed as previously described [28]. Nuclear and cytoplasmic fractions were prepared as previously described [47]. The NFκBp65 antibody was purchased from Cell Signaling Technology (Danvers, MA). Histone H3 (antibody purchased from Cell Signaling Technology) was used as loading control for the nuclear fraction, β-actin or α-tubulin for cytoplasm, and β-actin for total lysate. Signals were detected using the Odyssey Infrared Imaging System (LI-COR Biosciences, Lincoln, NE) and quantified using the Odyssey software (version 3.0; LI-COR Biosciences). Levels of ARC and phospho-NFκBp65 (p-NFκBp65) proteins in ARC KD and OE cells were also determined by CyTOF mass cytometry (Fluidigm, San Francisco, CA) after cells were stained with a panel of antibodies for cell surface markers and intracellular molecules as previously described [48]. ARC antibody (Santa Cruz Biotechnology, Inc.) was tagged with metal 170Er and p-NFκBp65 antibody (Cell Signaling Technology) with 171Yb.

The expression of cell surface CCR2, CCR5 and CXCR4 was determined by flow cytometry after cells were stained with their respective antibodies and IgG controls and results were expressed as relative mean fluorescence intensity (rMFI) determined by (MFI of antibody staining – MFI of IgG)/MFI of IgG. Antibodies against CCR2 and CCR5 were purchased from R & D Systems Inc. and CXCR4 from bioscience, Inc. (San Diego, CA).

The IL1β levels in culture media and mouse serum were determined by Quantikine human IL1β ELISA kit (R & D Systems Inc.) following manufacturer's instruction. The sera used were collected from NSG mice 3 weeks after injected with ARC KD or control Molm13 cells as previously described [28], and frozen at −20°C.

### Structural analysis of ARC

The compatibility of the ARC N-terminal sequence with CARD folds was assessed through three-dimensional structural modeling using iTasser (http://zhanglab.ccmb.med.umich.edu/I-TASSER/) and Swiss Model (http://swissmodel.expasy.org/) programs, based on 27–29% sequence identity with experimentally established structures of CARD domains (PDB accession numbers 2b1w, 2nz7 and 4jqw). Protein structural disorder was predicted using the PrDOS meta-server (http://prdos.hgc.jp/cgi-bin/top.cgi). ELM (http://elm.eu.org/) was used for prediction of function sites within the protein sequence. During publication of our study, the ARC CARD domain crystal structure was published (PDB entry 4UZ0) [33] corroborating our analysis.

### Statistical analyses

All experiments were conducted in triplicate and the results are expressed as mean ± standard deviation (error bars). Correlations are expressed as R^2^. Statistical differences between groups were determined using paired Student's *t*-test with a *P*-value < 0.05 being considered statistically significant. For determining protein expression using CyTOF mass cytometry, data were analyzed with viSNE software (v1.1, released February, 2015) and association maps were generated with all cell surface and intracellular markers [49].
